# Tailor‐made PAT platform for safe syngas fermentations in batch, fed‐batch and chemostat mode with *Rhodospirillum rubrum*

**DOI:** 10.1111/1751-7915.12727

**Published:** 2017-06-06

**Authors:** Stephanie Karmann, Stéphanie Follonier, Daniel Egger, Dirk Hebel, Sven Panke, Manfred Zinn

**Affiliations:** ^1^ Institute of Life Technologies University of Applied Sciences and Arts Western Switzerland (HES‐SO Valais) Sion Switzerland; ^2^ Department of Biosystems Science and Engineering ETH Zurich (ETHZ) Basel Switzerland; ^3^ Infors AG Bottmingen Switzerland

## Abstract

Recently, syngas has gained significant interest as renewable and sustainable feedstock, in particular for the biotechnological production of poly([*R*]‐3‐hydroxybutyrate) (PHB). PHB is a biodegradable, biocompatible polyester produced by some bacteria growing on the principal component of syngas, CO. However, working with syngas is challenging because of the CO toxicity and the explosion danger of H_2_, another main component of syngas. In addition, the bioprocess control needs specific monitoring tools and analytical methods that differ from standard fermentations. Here, we present a syngas fermentation platform with a focus on safety installations and process analytical technology (PAT) that serves as a basis to assess the physiology of the PHB‐producing bacterium *Rhodospirillum rubrum*. The platform includes (i) off‐gas analysis with an online quadrupole mass spectrometer to measure CO consumption and production rates of H_2_ and CO_2_, (ii) an at‐line flow cytometer to determine the total cell count and the intracellular PHB content and (iii) different online sensors, notably a redox sensor that is important to confirm that the culture conditions are suitable for the CO metabolization of *R. rubrum*. Furthermore, we present as first applications of the platform a fed‐batch and a chemostat process with *R. rubrum* for PHB production from syngas.

## Introduction

Municipal solid waste (MSW) is a complex mixture of waste from households or public institutions. Although almost constant since 2004, the annual amount of MSW produced in the European Union is substantial, with over 5 tons per person in 2014 (Eurostat, 2014) and leading to a yearly amount of 66 million tons ending up in landfills (Eurostat, 2014).

One possible way to harvest this resource for a circular economy is pyrolysis, a method where organic material is thermochemically decomposed in the absence of oxygen (Velghe *et al*., 2011). The gas fraction obtained from this process is called syngas, and it is composed of CO, H_2_ and CO_2_. It is of particular interest due to its potential as a cheap and renewable feedstock for (bio)chemical processes. Furthermore, syngas does not interfere with the food chain and no agricultural land and water are needed for its production. It is also available as a direct waste product from the steel industry (Molitor *et al*., 2016). Besides chemical catalysis (van de Loosdrecht and Niemantsverdriet, 2012; Mesters, 2016), also biological conversion is possible by some autotrophic bacteria that transform syngas into high‐value products including H_2_, methane, carboxylic acids (acetic and butyric acid), alcohols (ethanol, butanol) or esters (Munasinghe and Khanal, 2010; Bengelsdorf *et al*., 2013). In contrast to chemical catalysis, bacteria are less sensitive to changes in the CO to H_2_ ratio or traces of sulphur and chlorine in syngas (Mohammadi *et al*., 2011).

*Rhodospirillum rubrum*, a Gram‐negative, facultative photosynthetic, purple non‐sulphur bacterium is a versatile CO‐metabolizing bacterium. It can grow hetero‐ or autotrophically, aerobically as well as anaerobically. With the enzyme CO dehydrogenase (CODH) *R. rubrum* can oxidize CO with H_2_O and produce CO_2_ and H_2_ (water–gas shift reaction) under anaerobic conditions at a redox potential below −300 mV (Heo *et al*., 2001; Najafpour and Younesi, 2007; Younesi *et al*., 2008). At the same time, *R. rubrum* can intracellularly accumulate carbon as poly([*R*]‐3‐hydroxybutyrate) (PHB) (Kerby *et al*., 1992, 1995; Do *et al*., 2007). PHB is a biopolyester of the polyhydroxyalkanoate (PHA) family that is particularly interesting because of its polypropylene‐like properties, biodegradability and biocompatibility (Sudesh *et al*., 2000; Zinn *et al*., 2001). It has been shown that the PHB production on syngas reaches larger quantities when *R. rubrum* is cofed with acetate or malate. As an example, for cultures grown in serum vials with syngas as carbon source, the PHB content increased from 2 to 20 wt% when the acetate concentration in the medium was increased from 0 to 10 mM (Revelles *et al*., 2016).

To date, PHB from syngas has not been produced at industrial scale yet because of insufficient PHB productivities. To be able to perform a systematic process optimization with *R. rubrum* and get a detailed understanding of the cell physiology, a tailor‐made platform equipped with process analytical technology (PAT) is a necessity. PAT is part of the ‘quality by design’ guideline published by the American Food and Drug Administration (FDA). Its goal is to ensure the quality of a (pharmaceutical) product by a deep understanding of its production process (FDA, 2004). PAT includes real‐time analytical monitoring, interpretation and feedback controls by a computer or the operator. For syngas fermentations, it is mainly desirable to follow the consumption of the substrate CO (dissolved in the culture broth and in the off‐gas), the product formation (intracellular PHB and CO_2_ and H_2_ in the off‐gas) and the biomass production.

Biomass quantification is a key parameter in process monitoring and control, it can be determined either at‐line by measuring the optical density at 600 nm (OD_600_), in‐line with capacitance or optical probes (Kiviharju *et al*., 2007), or as total cell count (TCC) with flow cytometry (FCM). FCM gained importance for PAT with the increasing availability of at‐line methods and fully automated online systems (Abu‐Absi *et al*., 2003; Broger *et al*., 2011; Hammes *et al*., 2012). Furthermore, FCM can be used to quantify the intracellular PHB content in near real‐time (Kacmar *et al*., 2006; Lee *et al*., 2013; Karmann *et al*., 2016). A recently published staining protocol based on BODIPY493/503 and SYTO 62 for PHB‐DNA‐double staining ensures a complete recording of TCC including cells not containing PHB and the determination of the PHB content excluding false positives caused by cell debris or free PHB from lysed cells (Karmann *et al*., 2016).

Typically, at‐line or online gas chromatography (GC) is used to measure the syngas composition in the off‐gas (Do *et al*., 2007; Chipman, 2009; Revelles *et al*., 2016). However, quadrupole mass spectrometry (QMS) is more suitable for online analysis of fermentation off‐gases than GC as the measurement is faster (in the order of 1 min). Nevertheless, syngas is a complex mixture of gases and difficult to quantify, in particular because N_2_ and CO both have a molecular weight of 28. Therefore, a special analysis protocol is needed (Le *et al*., 2015).

Few syngas fermentation platforms have been described in literature in the last years. Do *et al*. (2007) presented a fermentation platform including an *in situ* syngas production facility for the gasification of corn and equipped with tools to remove char, tar and ash from the gas before sparging it into a bioreactor, where *R. rubrum* was grown to produce PHB. The fermentation was followed with real‐time off‐gas analysis based on GC, but biomass and PHB quantifications were performed offline and, in case of PHB, involved a lengthy extraction procedure. Some years later, a new platform was developed in the same laboratory, this time including parallel 14 l bioreactors and a remote monitoring and control panel programmed with LabVIEW (Chipman, 2009). An alarm system was set up to send a text message to the operator in case of overpressure and if deviations in the gas mass flow rates occurred.

For syngas fermentations, safety aspects have to be considered as large amounts of H_2_ and CO are continuously sparged into the bioreactor. H_2_ can be hazardous if not handled properly because of a high flammability (Table 1). Strong ultraviolet radiation is generated when it burns and destructive explosions are likely to occur in case ignition happens in a confined space. Being colourless, odourless, tasteless and burning with an almost invisible flame, H_2_ is difficult to detect. In addition, H_2_ molecules are extremely small and light, which makes them prone to leak. One should also note that, although not corrosive, H_2_ can alter the mechanical properties of metals such as steels, aluminium, titanium, nickel and their alloys (‘hydrogen embrittlement’) (Louthan *et al*., 1972).

**Table 1:** Characteristics of H_2_ and CO related to safety issues

	H_2_	CO
Flammability range in air	4.0–75.0 vol%^a^	12.5–74.2 vol%^b^
Auto‐ignition temperature in air	585°C^c^	605°C^b^
Specific gravity (compared to air at 20°C, 1 atm)	0.07^a^	0.97^b^
Permissible exposure limit (PEL)	n. a.	35 ppm^b^
Recommended exposure limit (REL)	n. a.	50 ppm^b^
Immediately dangerous to life or health value (IDLH)	n. a.	1200 ppm^b^

n. a. not available

a Zabetakis (1965).

b NIOSH (2016).

c White *et al*. (2006).

Like H_2_, CO is colourless, odourless, tasteless and flammable (Table 1). It is, however, much heavier than H_2_ and will mainly follow the air streams in case of leakage. The major hazard is its toxicity to humans, already in the ppm range. CO binds to haem proteins in human blood with a more than 200 times higher affinity than oxygen (Joels and Pugh, 1958), in particular to haemoglobin (Hb) thereby preventing oxygen transport. Exposure to 30–50 ppm CO will result in 5–8% COHb (of total Hb) and have adverse effects on healthy patients, while exposure to more than 600 ppm CO will increase the COHb up to 50%, the risk of death, caused by cardiac arrhythmia, is high (Dolan, 1985; ATSDR, 2012). As a result, exposure limits have been set by the safety institutions of most countries, for example by the U.S. National Institute for Occupational Safety and Health (NIOSH) and the Occupational Safety and Health Administration (Table 1).

In this article, we describe the syngas fermentation platform that we set up for the production of PHB by *R. rubrum* including (i) a series of safety measures for handling CO and H_2,_ and (ii) different PAT tools for monitoring the off‐gas as well as the production of biomass and PHB during fed‐batch and chemostat processes. This platform allowed the collection of particularly useful data for physiological studies that will build a basis to further improve the PHB productivity.

## Results

### Design of a safe laboratory platform

Because of the hazardousness of CO and H_2_, an appropriate laboratory environment is required for syngas fermentations. This includes specific elements to (i) prevent gas leakages, (ii) detect gas leaks and raise an alarm in case they occur, and (iii) deal with gas leaks in case of emergencies so as to avoid any negative effects on human health and material damage.

**Figure 1:**
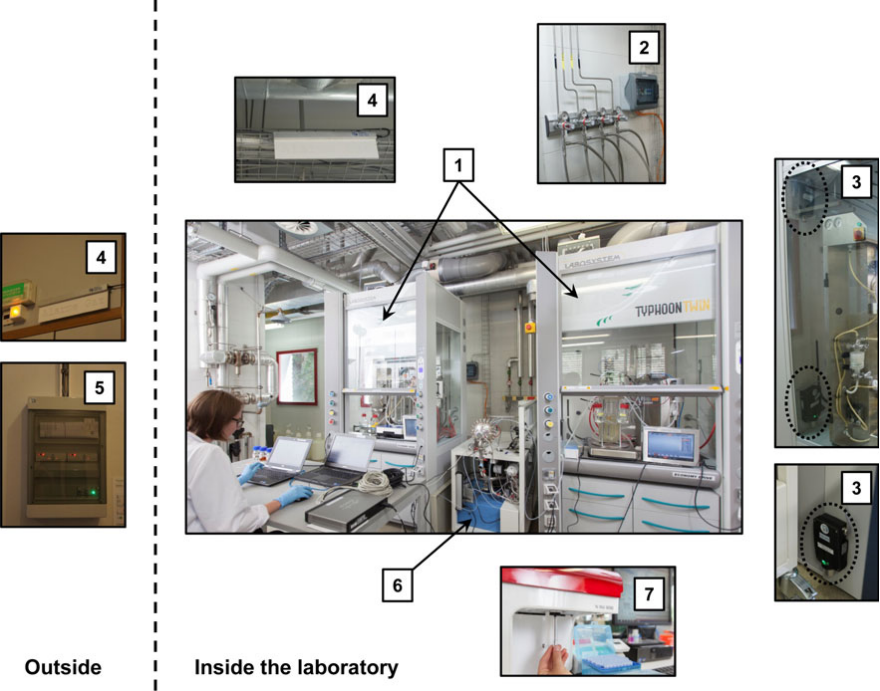
Safe PAT platform for syngas fermentations. Two fume hoods harbour each a bioreactor and ensure the rapid evacuation of H_2_ and CO in case of leakage (1). Gas pipes are used for the transport of H_2_, CO, CO_2_ and N_2_ from gas cylinders stored outside the building into the laboratory, and they are equipped with both safety shutoff electrovalves and manual pressure regulators (2). Detectors for H_2_ and CO are located inside each fume hood in the upper and lower part, respectively, according to the gas density. Because of the higher risk of CO, one additional detector is placed for this gas in the vicinity of the fume hoods (3). Visual and sound alarms are used to warn the personnel both inside and outside the laboratory in case of a problem (4). The concentrations of H_2_ and CO measured by the detectors are safely accessible on a control panel located outside the laboratory (5). A quadrupole mass spectrometer (QMS) continuously analyses the off‐gases during a bioprocess (6), and the total cell count as well as the intracellular PHB content are measured at‐line with a flow cytometer (7).

An overview of the safety measures that we designed specifically for bioprocesses with two Labfors 5 benchtop bioreactors (3.6 and 13 l) is given in Fig. 1. Specifically, fume hoods ensure low levels of H_2_ and CO in case of leakage, the gas cylinders are stored outside the building and safety shutoff electrovalves control the gas alimentation to the fume hoods. Detectors for CO and H_2_ with both visual and sound alarms are used to warn the personnel in case of elevated concentrations. The first alarm thresholds are set to 100 ppm and 0.8 vol% for CO and H_2_, respectively, and the second threshold to 150 ppm and 1.6 vol%. In case the second threshold is reached, the safety electrovalves are automatically closed, as do they if a power shortage occurs or if an emergency switch is triggered.

As an additional measure to prevent leakage, special care was taken to always use appropriate fittings including nuts, ferrules and tubing inserts for pipe and tubing connections. Furthermore, we selected as material for flexible tubings perfluoroalkoxy alkane (PFA), which exhibits similar properties as polytetrafluoroethylene and is resistant to high temperature and has a low gas permeability (Extrand and Monson, 2006).

### Development of a continuous off‐gas analysis method using mass spectrometry

We developed a fast and quantitative method to study the variation of H_2_, CO, CO_2_, N_2_ and H_2_O in the off‐gas based on mass spectrometry. This technique is very powerful, yet quantification can be challenging in case of complex mixtures like syngas. Indeed, syngas analysis results in the overlap of the masses of several gases (e.g. both CO and N_2_ have a mass to charge ratio m/z of 28) or fragments thereof (see Table S2, Supplementary data). The QMS software is able to distinguish the different gases by considering not only their molecular masses but also the masses of their fragments whose relative abundancy is fixed for given analysis settings. Nevertheless, accurate measurements can only be achieved if the analysis settings (voltage, electron emission, electron energy, dwell and settle times) are properly chosen and the system precisely calibrated (see Experimental procedures and Table S2). In addition, as the fragmentation pattern of the gases is critical for these analyses, we determined them experimentally under the same settings as for the off‐gas analysis instead of relying on the software library. The analysis settings were optimized until the measurements of both pure gases and syngas mixtures of known composition reached satisfactory results, that is a relative error below 8% (arbitrary threshold). An example of such a verification test is given in Supplementary data Table S1, showing a relative error of the measurements < 5%.

### Set‐up of a syngas fermentation platform for batch, fed‐batch and chemostat cultivations

**Figure 2:**
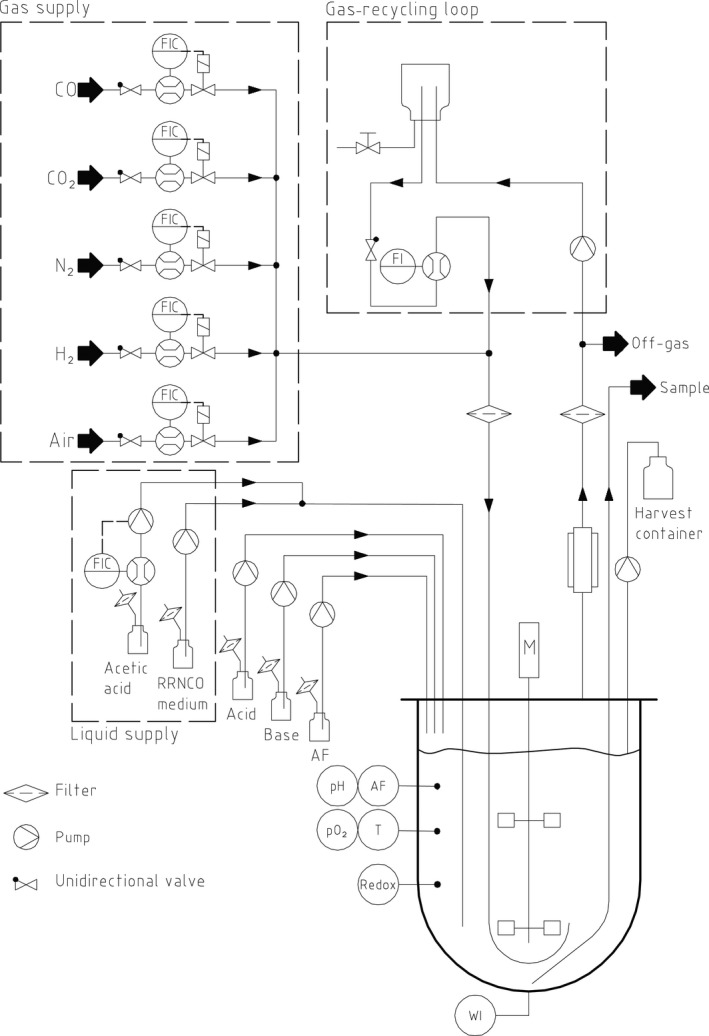
Schematic layout of the bioreactor for fed‐batch or chemostat cultivations on syngas. The gas supply is performed with five mass flow controllers (MFCs) shown as flow indicator controller (FIC) connected to different gas cylinders. The liquid supply consists of feed for acetic acid controlled with a liquid MFC and a feed for modified RRNCO medium. A gas‐recycling loop can be activated to reuse off‐gas with a high CO content. Online sensors are installed to measure and/or control pH, antifoam (AF), pO_2_, temperature (T), redox potential (Redox) and a balance (WI). The off‐gas passes a condenser and is then connected to the quadrupole mass spectrometer (QMS) for online off‐gas analysis. *Only in use for continuous cultivations.

We developed a versatile laboratory‐scale fermentation platform for the optimization of PHB production from syngas with *R. rubrum*. The set‐up for batch, fed‐batch and chemostat cultivations is shown in Fig. 2. It was possible to use either a 3.6 or 13 l bioreactor as the vessels were easily interchangeable. A custom‐made gas mixing station with five individual mass flow controllers (MFCs) enabled generating accurate mixtures of syngas. A liquid MFC allowed the precise feeding of a concentrated substrate (here acetic acid) for the fed‐batch phase. The solution was delivered directly in the culture broth with a metallic tube, which avoided irregular feeding by drop formation when applying very small flow rates. The bioreactors were equipped with standard sensors [pH and antifoam (AF)] and with a redox sensor providing online data. The off‐gas was passed through a condenser to remove as much H_2_O as possible before it reached the QMS. If necessary, part of the gas could be recycled using a gas‐recycling loop. A balance was placed underneath the culture vessel to estimate the current culture volume that was necessary to calculate the feed or dilution rate (see Experimental procedures).

In case of chemostat cultivation, a second pump for larger flow rates continuously transferred medium (here modified RRNCO medium) into the bioreactor. To keep the volume constant during continuous cultivation, the culture broth was pumped out into the harvest container using a metallic tube installed at a level corresponding to a chosen volume (Fig. 2). All data were recorded by the same software and could be built into automated feedback‐control loops.

### Fed‐batch fermentation with *R. rubrum* on syngas

**Figure 3:**
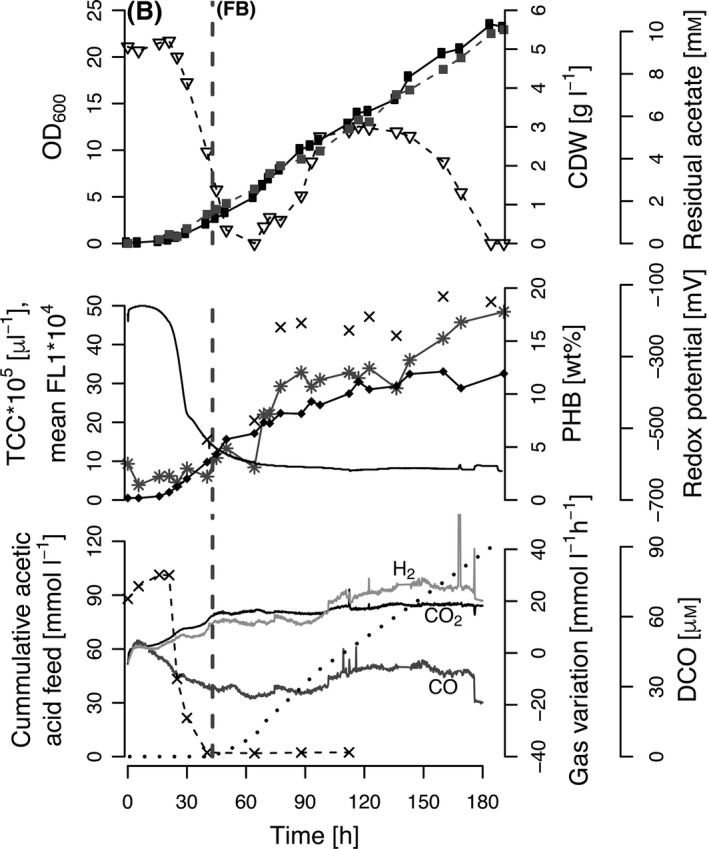
Fed‐batch fermentation with *Rhodospirillum rubrum* with continuous syngas aeration at 0.1 l l^−1^ min^−1^ and continuous feeding of acetic acid. The batch phase (B) and the fed‐batch phase (FB) are separated by a grey dashed line. The upper panel shows the culture optical density at 600 nm (• OD_600_), residual acetate (▽) and the cell dry weight (■ CDW). The middle panel displays the redox potential (‐), intracellular PHB content in wt% determined by GC (×), as well as the PHB content (✳ mean FL 1) and the total cell count (♦ TCC) both measured by flow cytometry. The lower panel shows the cumulative acetic acid feed (∙∙∙), the dissolved CO (× DCO) and the consumption and production rates of CO, H_2_ and CO_2_ as labelled in the plot.

The syngas fermentation platform was tested for *R. rubrum* in a fed‐batch fermentation starting with a batch phase on modified RRNCO medium supplemented with 10 mM acetic acid and a continuous syngas aeration at a specific gassing rate of 0.1 l l^−1^ min^−1^. The fed‐batch phase was started after 43 h with a continuous feed of acetic acid (see Experimental procedures), required to trigger PHB accumulation (Fig. 3). During the batch phase, the cells grew exponentially at maximal specific growth rate μ_max_ of 0.1 h^−1^ (based on the OD_600_) until CO limitation was reached. This limitation was indicated by the dissolved CO (DCO) concentration that reached non‐detectable values (< 2 μM) 43 h after inoculation (Fig. 3). The redox potential, which has been previously shown to influence the activity of the CODH (Heo *et al*., 2001), followed a similar trend and dropped from −160 mV at the time of inoculation to −550 mV at the end of the batch phase. The CO consumption and the H_2_ and CO_2_ production rates determined from the off‐gas measurements also reached a plateau at the end of the batch phase.

The PHB formation was followed by measuring the mean green fluorescence (mean FL1) of BODIPY 493/503 stained cells by FCM. Only a small amount of PHB, approximately 5%, was accumulated during the batch phase of the fermentation.

At the end of the fed‐batch (after 184 h of cultivation), an OD_600_ of 23.5 and a cell dry weight (CDW) of 5.5 g l^−1^ were reached. The PHB content increased during the fed‐batch phase and reached a final amount of almost 20 wt%. Interestingly, the TCC increased mainly during the first phase of the fed‐batch (until 110 h) and stayed constant, whereas the PHB content increased until the end. The redox potential remained constant at around −600 mV.

### Chemostat cultivation with *R. rubrum* on syngas

**Figure 4:**
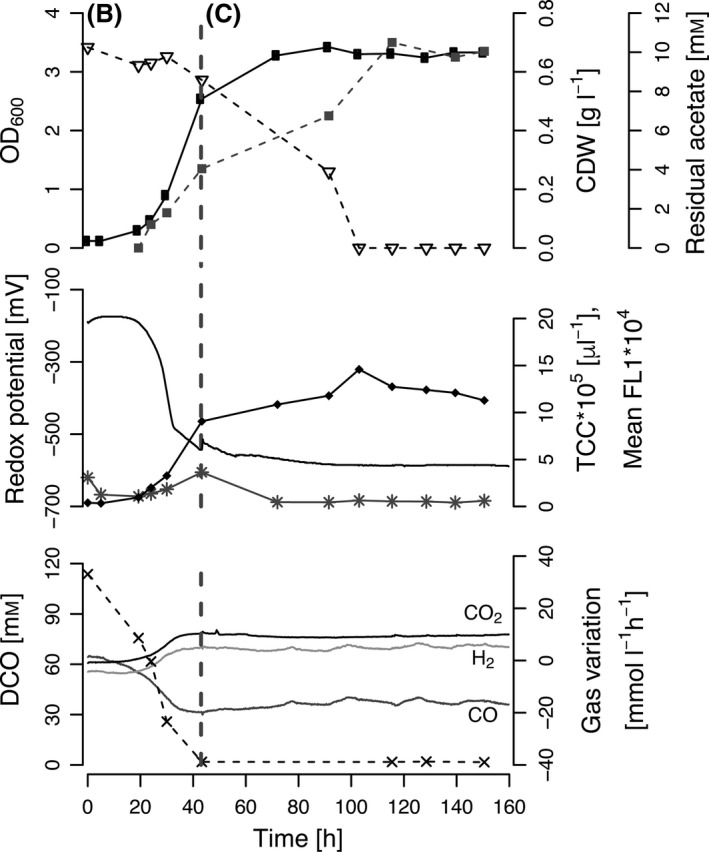
Continuous cultivation with *Rhodospirillum rubrum* on modified RRNCO medium containing 10 mM of acetate (*D* = 0.02 h^−1^) and a continuous syngas supply at 0.1 l l^−1^ min^−1^. The batch phase (B) and the chemostat phase (C) are separated by a grey dashed line. The upper panel contains biomass data with optical density at 600 nm (• OD_600_), the cell dry weight (■ CDW) and the acetate concentration in the culture supernatant (▽). The middle panel shows the redox potential (–), the PHB content (✳ mean FL 1) and the total cell count (♦ TCC) measured by flow cytometry. The lower panel contains the concentration of dissolved CO (× DCO) and the CO, H_2_ and CO_2_ consumption and production rates as labelled in the plot.

A continuous bioprocess was performed starting with the same batch phase conditions as for the fed‐batch described above. After 43 h, the continuous mode was started by constantly adding fresh modified RRNCO medium supplemented with 10 mM acetic acid at a dilution rate of *D* = 0.02 h^−1^. Syngas was constantly sparged at 0.1 l l^−1^ min^−1^ into the medium. After two volume changes (50 h), all measured parameters reached a plateau indicating the steady state was achieved. Under these conditions, the OD_600_ was stable at 3.3 ± 0.04, the CDW at 0.67 ± 0.02 g l^−1^ and the intracellular PHB content at 4.7 wt%, as determined by FCM. Also, the CO consumption rate and H_2_ and CO_2_ production rates were constant. The DCO was below the detection limit of 2 μM, indicating a CO mass transfer limitation. The redox potential stayed constant at less than −600 mV even though the culture was constantly provided with (aerobic) modified RRNCO medium that had not been purged with N_2_ or syngas (Fig. 4).

## Discussion

To date, syngas fermentation has been performed mainly at small‐scale in serum vials (Abubackar *et al*., 2012; Revelles *et al*., 2016). The advantage of this cultivation system is that it does not require a continuous syngas flow and therefore only limited safety measures are necessary. All manipulations can be simply accomplished in a fume hood. The gas tight vials can be incubated in regular incubators as no gas exchange with the environment is possible. However, a significant drawback of the serum vial cultivation technique is that only small growth rates of 0.02 h^−1^ and low final biomass concentration with an OD_600_ around 1 can be reached (Revelles *et al*., 2016). In addition, serum vials or shake flasks suffer from a low gas mass transfer and are rarely equipped with online sensors and consequently lead to a poor bioprocess understanding.

In this work, we set up a platform allowing to carry out syngas fermentation safely at larger scale while gaining a maximum of information about the bioprocess with specifically integrated PAT tools (redox sensor, QMS, FCM).

This platform was successfully applied to fed‐batch and chemostat cultivations of *R. rubrum* on syngas. As expected, *R. rubrum* reached higher cell densities in both types of bioprocesses than with the cultivations in serum vials mentioned above. The CDW and PHB accumulation differed significantly between fed‐batch and chemostat steady states (Figs 3 and 4). Due to the different amounts of total cosubstrate feeding, higher CDW and PHB values were found in the fed‐batch fermentation. Furthermore, in the chemostat steady state the cells were growing exponentially and therefore had to invest a significant portion of carbon into biomass formation, whereas during the fed‐batch cultivation, the cell number remained constant in the second half of the bioprocess (see TCC data in Fig. 3). Consequently, *R. rubrum* was able to use a bigger portion of carbon to synthesize PHB.

The online redox sensor confirmed that the redox potential in the medium was low enough (below −300 mV) to ensure the full activity of the CODH, the key enzyme of the CO metabolism of *R. rubrum* (Heo *et al*., 2001). A pO_2_ sensor would not be sensitive enough to give this information as a pO_2_ signal of 0.1% corresponds to a redox signal of up to −50 mV (Carius *et al*., 2013). The addition of a redox indicator such as resazurin to confirm anaerobic conditions was not required. Furthermore, no reducing agents like Na_2_S or cysteine‐HCl were necessary, even though aerobic acetic acid (fed‐batch) or modified RRNCO medium (chemostat cultivation) were fed. This is important to mention as resazurin and cysteine‐HCl have shown growth inhibiting effects on *R. rubrum* (Schultz and Weaver, 1982). Interestingly, the redox potential correlated very well with the DCO in the medium, especially during the batch phase (see Figs 3 and 4). This could potentially be implemented in the bioprocess control to rapidly detect CO limitation and replace the time‐consuming at‐line DCO quantification with myoglobin.

The CO content in the off‐gas, determined with the QMS, stands in agreement with the DCO data that also reached a plateau after the switch to the fed‐batch and the chemostat phase respectively (Figs 3 and 4). CO consumption and H_2_ and CO_2_ production rates increased in parallel. However, there is a shift in the off‐gas variation during the fed‐batch process at 112 h of cultivation that does not follow the theory where a smaller CO consumption rate is expected to lead to decreased H_2_ and CO_2_ production rates (Fig. 3). We realized that the off‐gas filter was blocked due to too much humidity in the off‐gas. This humid filter caused an overpressure in the bioreactor that presumably had an effect on the accuracy of the off‐gas measurement. This problem can be solved by exchanging the off‐gas condenser with a more powerful one. In general, the measurement of CO and CO_2_ in the off‐gas is necessary to establish carbon balances and calculate CO‐based growth and PHB yields. Furthermore, the quantification of the H_2_ production is also important as it can be sold as valuable by‐product.

FCM is another flexible tool of the PAT platform. The PHB quantification by FCM needs much less time and sample volume than the GC method that requires 5–50 mg freeze‐dried biomass, depending on the protocol (Brandl *et al*., 1988; Furrer *et al*., 2007). The PHB quantification by FCM has been shown to be at least as accurate as PHB quantification by GC (Karmann *et al*., 2016). In addition, during the fed‐batch fermentation presented here (Fig. 3), the data obtained by the two quantification techniques correlated very well (*R* ^2^ = 0.98, supplementary data Fig. S2). However, care has to be taken at the very end of the process as stationary phase cells can have different staining properties than exponentially growing cells (Karmann *et al*., 2016), and for very small PHB contents (< 5 wt%) where the intercept of the correlation equation could introduce a small error (supplementary data Fig. S2).

In contrast to CDW, OD_600_ or measurements by in‐line probes, the TCC determined by FCM provides information on whether the cells are dividing or whether the increasing biomass is due to intracellular PHB accumulation. As mentioned above, in Fig. 3 we can see clearly that the TCC is only increasing to a minor extent after 110 h of cultivation while the OD_600_ and CDW are still increasing continuously. This confirms that the latter measurements are influenced by PHB accumulation and thus do not provide accurate information about biomass in terms of the cell number.

Online sensors, especially temperature, pO_2_ and pH sensors are part of the standard equipment of a bioreactor. In case of syngas fermentation, we showed the advantage of a redox sensor, an online QMS and an at‐line FCM. All data from online measurements were collected by the same software and could be integrated in any feedback‐control loop. For example, if the redox potential suggests a complete consumption of DCO, an automated increase in syngas flow rate or a higher agitation speed could be implemented to increase CO mass transfer. In contrast, if the QMS indicates a poor consumption of CO as it is the case at the beginning of the fermentation process, a pump in the gas‐recycling loop could be switched on automatically and pump the CO‐rich off‐gas back into the culture vessel to avoid a waste of substrate (Fig. 2).

In conclusion, we presented here for the first time a safe laboratory‐scale fermentation platform that is tailor‐made for the production of PHB from syngas. We showed two applications of this PAT platform for syngas fermentation with *R. rubrum*, namely a fed‐batch and a chemostat cultivation process. On one hand, with the fed‐batch cultivation we showed that the PHB accumulation increased up to 20wt% when continuously feeding acetic acid as cosubstrate. On the other hand, chemostat cultivation is the technique of choice for process optimization as the influence of single cultivation parameters such as medium and syngas composition, pH, temperature and many more can be assessed individually in a very controlled and reproducible way. This is necessary for a better understanding of cell physiology to finally increase the PHB production from syngas. With only a few adaptations, the PAT platform for syngas fermentation can also be applied to biosynthesize a large variety of chemicals on other dangerous gas mixtures such as oxyhydrogen or methane. The QMS as well as the FCM are nearly unlimited in possible applications.

## Experimental procedures

### Safety installations

Two fume hoods (Typhoon twin, Labosystem, Rovellasca, Italy) were harbouring, each, a bioreactor and a cleaning‐ and sterilization‐in‐place unit (LabCIP; Infors AG, Bottmingen, Switzerland). Safety detectors for H_2_ and CO (Models EC28 and CC28, respectively, GfG AG, Binz, Switzerland) were located inside each fume hood, and one detector for CO was located outside but in the vicinity of the fume hoods. The sound and visual alarms (inside and outside the laboratory) as well as the control panel outside the laboratory were acquired from GfG AG (Binz, Switzerland). Gas pipes for H_2_, CO, CO_2_ and N_2_ (PanGas, Dagmersellen, Switzerland) were installed to transport the respective gases from cylinders stored outside the building into the laboratory and equipped with automatic shut‐off electrovalves (Model 21X2KV; ODE S. r. l., Colico, Italy). The pipes were connected to semi‐flexible PFA tubings (BGB Analytik AG, Boeckten, Switzerland) with appropriate fittings including nuts, ferrules and tubing inserts (Swagelok, Solon, United States).

### Bacterial strain, growth conditions and media

*Rhodospirillum rubrum* S1(ATCC 11170), stored in 1.5 or 15 ml 16% glycerol stocks (OD_600_ = 3.33) at −80°C, was used for all experiments. The liquid medium for chemostat and fed‐batch cultivations was a slightly modified version of the RRNCO medium (Kerby *et al*., 1995). It contained per litre of deionized water: 250 mg of MgSO_4_∙7H_2_O, 132 mg of CaCl_2_∙2H_2_O, 1 g of NH_4_Cl, 2.1 g of MOPS buffer, 1.0 g of yeast extract, 20 μM NiSO_4_, 10 ml of a chelated iron‐molybdenum solution (0.28 g of H_3_BO_3_, 2 g of Na_2_EDTA, 0.4 g of FeSO_4_ and 0.1 g of Na_2_MoO_4_ per litre of distilled water) and 2 μg of biotin. The medium was either sterile filtered (0.2 μm, Sartobran 300; Sartorius Stedim Biotech GmbH, Göttingen, Germany) or autoclaved except biotin and acetate which were always sterile filtered (0.22 μm filter; Sarstedt, Nümbrecht, Germany).

Precultures were grown in screw capped 1‐l bottles (Müller + Krempel AG, Bülach, Switzerland) filled with 500 ml of sterile filtered modified RRNCO medium at pH 7 (0.22 μm filter; Sarstedt) containing 15 mM fructose. The precultures were inoculated directly with 12 ml glycerol stock to reach an OD_600_ of 0.08 and were then incubated at 30°C on an orbital shaker at 180 min^−1^ for 46–48 h. During this time, gas exchange with the aerobic environment was impossible and anaerobic conditions were reached after exhaustion of O_2_ due to cell growth.

### Bioreactor settings for fed‐batch fermentation

The fed‐batch cultivation was carried out in a 13‐l benchtop bioreactor (Labfors 5; Infors AG) supervised by the supervisory control and data acquisition software Iris (Infors AG). The initial working volume was 10 l of autoclaved modified RRNCO medium supplemented with 10 mM acetate and 0.1 ml l^−1^ PPG2000 AF. The bioreactor was equipped with online sensors recording pH and redox potential (Hamilton Bonaduz AG, Bonaduz, Switzerland). The bioreactor was purged with syngas (25% CO, 25% H_2_, 5% CO_2_ and 45% N_2_) at 0.1 l l^−1^ min^−1^ for at least one hour before inoculation to reach an OD_600_ of 0.1.

The pH in the culture broth was maintained at 7.0 ± 0.05 by automated addition of 4 M KOH or 3 M H_2_SO_4_. The temperature was kept constant at 30°C, and the culture was mixed with two six‐blade Rushton impellers at 600 rpm. Syngas (25% CO, 25% H_2_, 5% CO_2_, 45% N_2_) was sparged continuously into the culture through a sinter filter sparger at 0.1 l l^−1^ min^−1^.

The batch phase was terminated after 43 h by starting the continuous feeding from a 2 M acetic acid stock solution with a pump controlled by a liquid MFC (mini Cori Flow M12; Bronkhorst Cori‐Tech B.V., Ruurlo, the Netherlands). The feed rate was adjusted twice per day based on OD_600_ measurements as follows: As long as the acetate in the culture supernatant was consumed completely, it was calculated based on the empirically determined equation 0.17 mmol OD_600_^−1^ l^−1^ h^−1^. The culture volume was measured with a balance (Kern KMB‐TM; Kern & Sohn GmbH) placed under the reactor. As soon as acetate accumulated in the medium, the feed rate was kept constant or reduced based on actual acetate consumption rates measured during the process.

### Bioreactor settings for chemostat cultivation

The chemostat cultivation was carried out in a 3.6 l benchtop bioreactor (Labfors 5; Infors AG) with a constant working volume of 2 l. The batch phase was terminated after 43 h by starting the continuous feeding of sterile filtered modified RRNCO medium supplemented with 0.1 ml l^−1^ PPG2000 AF for a dilution rate *D* = 0.02 h^−1^. Acetic acid was added separately at 0.2 g h^−1^ from a 2 M stock solution (final concentration of 10 mM in the total feed medium) (Fig. 2). The working volume was kept constant by continuously pumping culture through a harvest pipe installed at the volume of 2 l.

Samples were taken regularly during the bioprocesses. Analytics for biomass quantification, FCM and high pressure liquid chromatography (HPLC) and DCO were performed immediately. Pellets of biomass samples required for GC analysis were frozen at −80°C and treated as indicated below.

### Biomass quantification

Optical quantification of the biomass was performed spectrophotometrically at 600 nm (Libra S12; Biochrom, Cambridge, UK). For the measurement of the CDW, triplicates of 2 ml of cell culture (or suspension) were centrifuged in Eppendorf tubes (30,670 g, 4°C, 5 min), washed once with 1 ml 0.9% aqueous NaCl and then dried for 24 h in a 100°C oven. The tubes were then cooled down to room temperature in a desiccator and the CDW determined by subtracting the tare determined in advance.

### FCM analysis for PHB and TCC determination

Fresh culture samples were analysed in duplicates with a BD Accuri C6 flow cytometer (FCM) (BD Bioscience, Erembodegern, Belgium) and a BODIPY 493/503‐SYTO 62 double staining. The staining protocol published previously (Karmann *et al*., 2016) was adjusted slightly by increasing the incubation time to 30 min. This was due to the very slow growth rates especially at the end of the fed‐batch process and during the phase of continuous cultivation. The influence of the growth phase (exponential or stationary) on the staining kinetics has been addressed recently by Karmann *et al*. (2016) because it is known that cell envelope properties change based on the growth phase (Wanner and Egli, 1990).

During the chemostat fermentation, PHB was only quantified by FCM. The PHB content in wt% was calculated based the correlation established during the fed‐batch fermentation as shown in Supplementary data Fig. S2.

### Determination of residual acetate by HPLC

The acetate concentration in the culture supernatant was determined by HPLC (1100 Series; Agilent, Santa Clara, USA) equipped with a 300 mm × 7.8 mm ion exchange column (Aminex^®^ HPX‐87H; Bio‐Rad Laboratories, Hercules, USA). An isocratic elution was performed with 5 mM H_2_SO_4_ at a flow rate of 0.6 ml min^−1^ at 50°C for 30 min. Acetate was detected with a refractive index detector.

### PHB quantification by GC

For the PHB quantification by GC, cell pellets from 30 to 50 ml culture (3,900 g, 4°C, 10 min) were harvested during the fed‐batch fermentation and washed once with 0.9% aqueous NaCl, stored at −80°C for at least 24 h and freeze‐dried at −80°C at a pressure of 0.25 mbar (Cryodos, Telstar, Terrassa, Spain). Methanolysis of 10–15 mg freeze‐dried biomass was performed based on a previously published protocol with small modifications (Brandl *et al*., 1988): The biomass was mixed in a screw cap tube with 2 ml of methanol 15 vol% in H_2_SO_4_ and 2 ml of CH_2_Cl_2_ containing 2.5 g l^−1^ 2‐ethyl‐2‐hydroxybutyric acid as internal standard. Purified bacterial PHB and 3‐hydroxypentanoic acid (eNovation Chemicals, China) were used as external standards. The solution was incubated at 100°C for 3 h and then extracted in two steps with 2 ml dH_2_O. The solvent phase was dried with anhydrous Na_2_CO_3_ and Na_2_SO_4_ and was subsequently analysed by GC with an Agilent 6890 GC‐FID (G1530 A; Agilent) equipped with a BGD‐Wax column (30 m∙0.25 mm, film thickness 0.25 μm) (BGB Analytik, Böckten, Switzerland). The injection volume was 1 μl, the split ratio was 1:10 and the injection temperature was 250°C. The temperature was increased from 80°C to 240°C at a rate of 10°C min^−1^, and 3 ml min^−1^ helium was used as carrier gas. A flame ionization detector at 285°C was used for detection.

### DCO and off‐gas quantification

The DCO was quantified based on an absorbance test using myoglobin as described earlier (Jones, 2007). The myoglobin solution was prepared without the dialysis step. Tests in our laboratory showed that this step did not improve the analysis.

The off‐gas concentrations were quantified with a QIC Biostream quadrupole mass spectrometer and the data analysed using the QGA FT software (Hiden Analytical, Werrington, UK). Notably, N_2_ was continuously flushed into the purge inlet (~150 ml min^−1^) to avoid the accumulation of hazardous H_2_ in the QMS.

All gases were measured with a Faraday detector and with a voltage, electron emission and electron energy of 830 V, 100 μA and 70 eV respectively. Seven gases were considered for the analysis, namely H_2_, CO, CO_2_, N_2_, H_2_O, O_2_ and Ar. The measurement parameters for each gas are given in Table S2 (see Supplementary data). The background values for each gas except Ar were determined after flushing the QMS with pure Ar. Thereafter, calibration was performed with ambient air [from which the H_2_O concentration was calculated from the room temperature, pressure and relative humidity using the Buck equation (Buck, 1981)], as well as with a CO_2_/O_2_/N_2_ gas cylinder and/or with syngas (CO, CO_2_, H_2_, N_2_) artificially prepared with the highly accurate MFCs. All gases were purchased from PanGas (Dagmersellen, Switzerland). The calibration values were normalized with respect to N_2_, which was present in the three calibration gases. The accuracy of the measurement was verified with gases of known composition before analysis, as shown in Supplementary data Fig. S1. The analysis time was in the range of 1 min.

The CO consumption rate *n*_CO_ in mol min^−1^ was calculated using N_2_ as inert gas with the following equation (1)

(1) $$n_{\mathrm{CO}}=\frac{\frac{F_{G}}{V_{m}}\left(X_{{\mathrm{CO}}_{\mathrm{out}}}\frac{X_{N_{2}in}}{X_{N_{2}out}}-X_{{\mathrm{CO}}_{\mathrm{in}}}\right)}{100},$$

where *F*_G_ is the total gas volume flow in ml min^−1^, *V*_m_ the molar volume at 25°C in ml mol^−1^, $X_{{\mathrm{CO}}_{\mathrm{out}}}$ the CO content in mol % in the off‐gas measured by MS, $X_{N_{2\mathrm{in}}}$ the N_2_ portion in the syngas feed in mol %, $X_{N_{2\mathrm{out}}}$ the N_2_ content in mol % in the off‐gas measured by MS, and $X_{{\mathrm{CO}}_{\mathrm{in}}}$ is the CO portion in syngas feed in mol %. The H_2_ and CO_2_ production rates ($n_{H_{2}}\;\text{and}\;n_{{\mathrm{CO}}_{2}}\;$) were calculated accordingly using the modified versions of Eq. 1 by replacing CO‐related parameters by H_2_ and CO_2_ ones, respectively.

## Conflict of Interest

Stephanie Karmann, Stéphanie Follonier, Sven Panke and Manfred Zinn declare that they have no conflict of interest. Daniel Egger and Dirk Hebel disclose that they are employed by Infors AG, the manufacturer of the bioreactors described in this manuscript.
